# Sustained response to pembrolizumab in recurrent perivascular epithelioid cell tumor with elevated expression of programmed death ligand: a case report

**DOI:** 10.1186/s13256-021-02997-x

**Published:** 2021-07-24

**Authors:** Ali McBride, Andrew J. Garcia, Lauren J. Sanders, Kelly Yiu, Lee D. Cranmer, Phillip H. Kuo, Matthew Kay, Andrew S. Kraft

**Affiliations:** 1 University of Arizona Cancer Center, 1515 N. Campbell Ave., Tucson, AZ 85718 USA; 2 College of Pharmacy, University of Arizona, Tucson, AZ USA; 3 College of Medicine, George Washington University, Washington, DC USA; 4 Department of Medical Imaging, Medicine and Biomedical Engineering, University of Arizona College of Medicine, Tucson, AZ USA; 5 Seattle Cancer Care Alliance, Seattle, WA USA

**Keywords:** PEComa, PD-L1, Pembrolizumab

## Abstract

**Background:**

Perivascular epithelioid cell tumors are defined by the World Health Organization as “a collection of rare mesenchymal tumors composed of histologically and immunohistochemically distinctive perivascular epithelioid cells.” Whereas localized perivascular epithelioid cell tumor is typically benign and treated successfully with surgical resection, prognosis for patients with advanced or metastatic perivascular epithelioid cell tumor is unfavorable, and there is no standard curative treatment.

**Case presentation:**

We report a Caucasian case of metastatic perivascular epithelioid cell tumor previously treated with chemotherapy and surgery with elevated surface expression of programmed cell death ligand 1. Based on this result, treatment via immune checkpoint inhibition with the monoclonal antibody pembrolizumab was pursued. After 21 cycles, the patient sustained a complete response. Therapy was stopped after the 40th cycle, and she was moved to surveillance. She remained disease free 19 months off treatment.

**Conclusions:**

This case report of a patient with perivascular epithelioid cell tumor treated successfully with programmed cell death protein-1 targeted therapy suggests that programmed cell death ligand-1 levels should be measured in patients with perivascular epithelioid cell tumor and immunotherapy considered for recurrent or metastatic patients. Future phase II/III studies in this disease should focus on sequencing of surgery and immunotherapy with a design of curative intent.

## Background

Perivascular epithelial cell tumors (PEComas) are an exceedingly rare, heterogeneous category of neoplasms identified in the last two decades [1]. In 1991, Pea *et al*. [2] were the first to describe perivascular epithelioid cell tumors in both angiomyolipomas (AML) and clear cell “sugar” tumors of the lung (CCST). A few years later, in 1996, Zamboni *et al*. [1, 2] introduced the name PEComa in their study of clear cell “sugar” tumors. This family of tumors, the etiology of which has yet to be elucidated, includes angiomyolipomas (AML), clear cell “sugar” tumors of the lung (CCST), lymphangioleiomyomatosis (LAM), and clear cell myomelanocytic tumors of the falciform ligament/ligamentum teres (CCMMT), as well as unusual clear cell tumors of the pancreas, rectum, abdominal serosa, vulva, uterus, heart, and thigh [1, 3, 4].

Reports of PEComa are observed to predominantly occur in women over a wide age range, with the exception of CCMMT, which commonly occurs in young girls with a mean age of 11 years at diagnosis. The most frequent reported sites of occurrence for PEComa are the kidney and uterus [5]. Typically, PEComa presents as a painless mass, with the exception of uterine PEComas and CCMMT, which present with vaginal bleeding and painful abdominal masses, respectively.

PEComas are characterized by perivascular location with a radial cell arrangement around the vascular lumen, involving epithelioid and spindled cells resembling smooth muscle that vary considerably by proportion between cases. Clear-to-mildly-eosinophilic cytoplasm and round-to-oval nuclei surrounded by small nucleoli are common, although nuclear irregularity has been observed [6]. PEComas typically display a myomelanocytic phenotype with expression of melanocytic markers, such as HMB-45, melan-A, and microphthalmia-associated transcription factor (MiTF), as well as myoid markers, such as smooth muscle actin (SMA) and caldesmon [3, 7]. Frequently, they are S100 and desmin negative [3].

PEComas are uniquely challenging cases not only diagnostically secondary to their diverse features and varying degrees of malignancy but also in terms of developing treatment approaches in advanced metastatic disease. Treatment of PEComa may be multimodal, potentially including chemotherapy (neoadjuvant and/or adjuvant), surgical resection, and radiation therapy. PEComas may or may not be associated with tuberous sclerosis complex (TSC) mutations. Studies have investigated whether loss of heterozygosity at TSC2 may be involved in the carcinogenesis of PEComa [5, 8–10]. These mutations on TSC1 and TSC2 inhibit the mechanistic target of rapamycin (mTOR) pathway, thereby leading to cell growth and proliferation. Indeed, current case report literature has demonstrated some utility in treatment of PEComas with mTOR inhibitors, such as rapamycin and sirolimus, which function to inhibit mTOR, which, as aforementioned, is activated in PEComas [5, 11, 12]. However, to date, no large-scale clinical studies have evaluated the use of mTOR inhibitors for PEComa, although preliminary data are promising and suggest the possibility of complete response [12, 13]. This outcome indicates the need for the development of new treatment options [11].

In the last few years, strides have been made in exploring PD-1/PD-L1 inhibition in the treatment of soft-tissue sarcomas upon recognition of increased expression of these markers in these tumors. In 2016, a study by Paydas *et al*. [14] noted that expression in soft-tissue sarcoma subtypes ranged from 20% to 66% for PD-1 and 25% to 33% for PD-L1. Although this study of 65 patients did not detail whether these levels were clinically meaningful or affected potential patient outcomes, it did suggest that treatment targeted at this pathway might be considered in these diseases. In their study of 82 soft-tissue sarcoma patients, Kim *et al*. [15] demonstrated 43% overall PD-L1 expression among the subtypes as follows: epithelioid sarcoma (100%), synovial sarcoma (33%), rhabdomyosarcoma (38%), Ewing sarcoma (33%), and mesenchymal chondrosarcoma (0%).

Having observed increased PD-L1 expression in sarcoma, clinical trials have examined targeting this pathway. In a phase II SARC028 trial in 2017, Tawbi *et al*. [16, 17] investigated objective response to pembrolizumab in 40 metastatic or surgically unresectable, local advanced, soft-tissue sarcoma patients having failed previous systemic antineoplastic therapy. Overall response, which was the primary endpoint, was not achieved. However, clinical response was observed with 18% objective response rate (ORR) and 12-week progression-free survival (PFS) of 55% in the soft-tissue cohort, suggesting some utility in undifferentiated pleomorphic sarcoma or dedifferentiated liposarcoma [16, 17]. In the Alliance A091401 phase II study of 85 metastatic sarcoma patients, D’Angelo *et al*. [18] similarly demonstrated the efficacy of anti-PD1 therapy with nivolumab, an anti-PD-1 monoclonal antibody, and ipilimumab, an anti-CTLA-4 monoclonal antibody. Treatment with nivolumab alone demonstrated minimal activity, with an objective response in 5% of patients and confirmed median PFS and overall survival benefit of 1.7 months and of 10.7 months, respectively [19]. Together, these results suggested that, although the majority of patients did not respond to this therapy, individual patients with specific disease phenotypes could be responders. Our current report highlights a patient initially diagnosed as having sarcoma but found to be histologically consistent with abdominopelvic sarcoma of perivascular epithelial cells (PEComa). The patient had immunohistochemistry showing elevated levels of PD-L1 and was successfully treated with pembrolizumab for their PEComa diagnosis.

## Case presentation

**Fig. 1:**
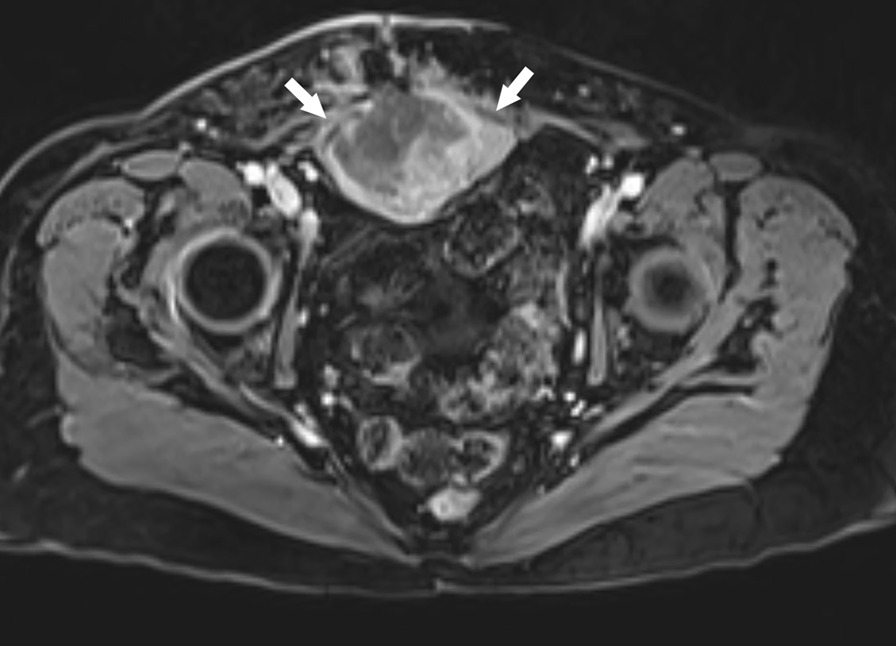
Axial T1 weighted post-contrast magnetic resonance imaging (MRI) demonstrating a large heterogeneously enhancing mass arising from the deep surface of the lower rectus abdominis muscle (arrows)

**Fig. 2:**
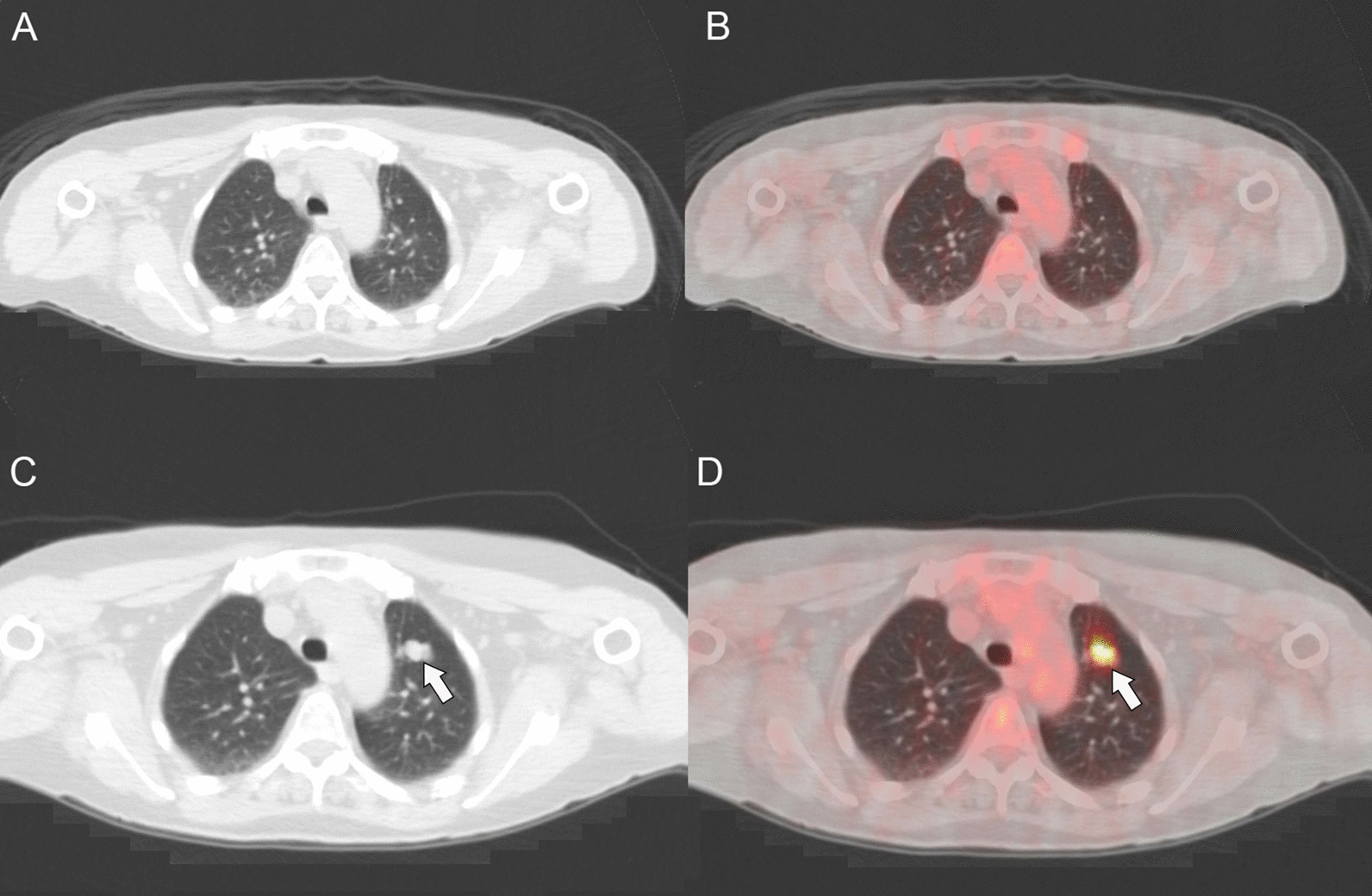
The CT (**a**) and PET/CT (**b**) performed November 2013 do not demonstrate any metabolically active metastases or pulmonary nodules. The subsequent CT (**c**) and PET/CT (**d**) demonstrate a metabolically active pulmonary nodule in the anterior segment of the left upper lobe (arrows), consistent with a pulmonary metastasis

The patient is a 69-year-old Caucasian female who reported asymmetry of her abdomen in early 2011. An abdominal mass was visualized with magnetic resonance imaging (MRI) (Fig. 1). CT-guided biopsy was undertaken, and pathology was consistent with a T2b grade 3 (stage IIB) high-grade soft-tissue sarcoma arising from the lower rectus abdominis muscle. In March 2011, following surgical resection with concurrent interstitial high-dose-rate brachytherapy, she had external beam radiation therapy (XBRT). The patient was observed, and 2 years later a solitary metastatic lesion to the anterior lingula of the left lung was noted on follow-up CT scan. This mass was resected in May 2013. Follow up PET/CT in August and November 2013 revealed no further evidence of disease other than a pulmonary nodule in the apical left upper lobe that was not fluorodeoxyglucose (FDG) avid. After an observational period of nearly 2 years, follow-up CT scan of the chest, abdomen, and pelvis confirmed stability of the apical left upper lobe pulmonary nodule. However, there was development of a pulmonary nodule more caudally in the anterior segment of the left upper lobe concerning for a possible solitary lung metastasis. The anterior segment left upper lobe pulmonary nodule had enlarged on the subsequent, March 2015 PET/CT scan, measuring 1.8 × 1.1 cm and was metabolically active, having a standardized uptake value (SUV) of 5.9, which was consistent with solitary pulmonary metastasis (Fig. 2). The patient received a left upper lobectomy in late March 2015. The specimen pathology was found to be metastatic PEComa and was sent for additional genetic testing.

**Fig. 3:**
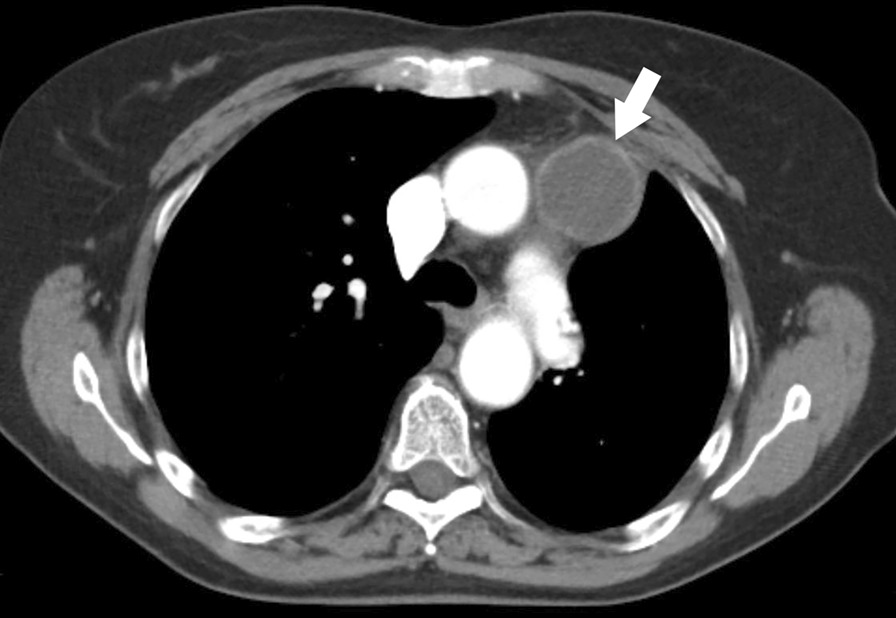
CT demonstrates metastatic disease involving a peripherally enhancing and centrally necrotic subaortic lymph node (arrow)

In September 2015, CT revealed progression of disease with development of a subaortic lymph node metastasis, in the anterior mediastinum (Fig. 3). In this case, surgical resection was deemed to not be a viable option. Therefore, systemic therapeutic strategies were instead considered. mTOR inhibitors were a potential choice of therapy, based on studies indicating some efficacy in this setting, but no clinical trials utilizing these agents were available [5, 11, 13, 20, 21]. Secondary to elevated expression of PD-L1 on IHC of the tumor, treatment with the PD-1 inhibitor pembrolizumab 2 mg/kg intravenously every 3 weeks was begun in October 2015.

**Fig. 4:**
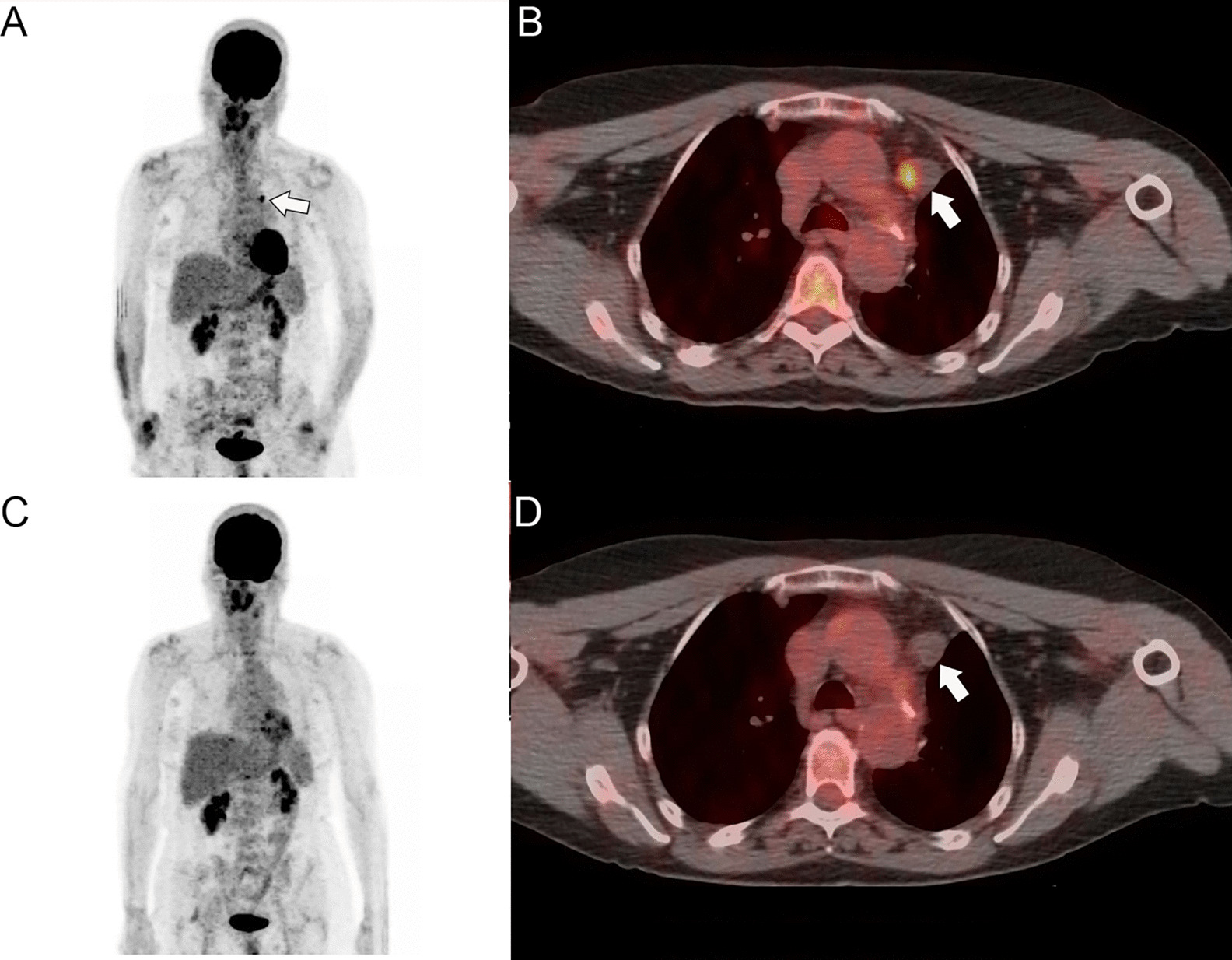
The PET (**a**) and PET/CT (**b**) performed at the time of recurrence in September 2015 demonstrate a metabolically active subaortic lymph node metastasis (arrows). The subsequent PET (**c**), performed approximately 1 year after commencing treatment with pembrolizumab, did not demonstrated any metabolically active disease, and the PET/CT (**d**) confirms no metabolic activity is associated with the prior subaortic lymph node (arrow)

After cycle 21, a little more than 1 year after commencing pembrolizumab therapy, the patient had a PET/CT scan with no metabolically active disease (Fig. 4). At this time, the patient’s only complaint was shortness of breath on exercise, which was attributed to the prior lung resection. She continued on pembrolizumab treatment with no evidence of disease until April 2018 for a total of 40 consecutive cycles at which time she was placed under surveillance. She has been disease free off therapy for 19 months.

## Discussion

A classification system was proposed by Folpe *et al*. [3] delineating benign, uncertain malignant potential, and malignant PEComas based upon the following so-called worrisome features: size > 5 cm, infiltrative border, high nuclear grade and cellularity, mitotic rate of 1 per 50 high-power fields or higher, necrosis, and vascular invasion [3]. Benign PEComas do not have these features, whereas potentially malignant potential PEComas possess size > 5 cm only or nuclear pleomorphism/multinucleated giant cells only and malignant PEComas possess two or more worrisome features. Although the degree of malignancy varies widely, most cases of PEComa are benign [3]. Our patient demonstrated widely metastatic and recurrent disease that was not cured by surgery and had a metastasis that was not easily approachable by this modality. She was found to have elevated levels of PD-L1 and started on immunotherapy with pembrolizumab. Her response was not immediate but took a number of months for a complete response (CR) to develop. It was not clear how long to keep her on therapy, but the patient elected to continue treatment for almost 2 years after this response.

While this patient was being treated, a case report described a patient with epithelioid angiomyolipoma who had failed everolimus even with a mutation in *TSC2* but had an elevated level of PD-L1 [22]. With highly metastatic disease, he was treated successfully with nivolumab, a PD-1 inhibitor. This patient had a response after five cycles and continued on therapy for 2 years. Like our patient, this individual had prolonged treatment with this immunotherapy. At this time, in PEComa patients who are responding, it is not clear how long therapy should be continued. Also, it is not known how this therapy should be sequenced with surgery. Initial upfront treatment with immunotherapy could save these patients significant morbidity from wide surgical resections. The use of tumor PD-L1 levels as a marker of those who will respond to immunotherapy is controversial. In the current and prior case reports, each of these patients had elevated levels of PD-L1, suggesting that in responders to this therapy these levels are helpful in treatment decisions in PEComas.

## Conclusions

We report here on a case of metastatic PEComa in a 69-year-old female, previously treated with chemotherapy and surgery with noted expression of PD-L1. Given the favorable outcome observed, in the case of recurrence following surgical resection of the primary tumor, PD-1/PD-L1 should be measured and the possibility of utilizing targeted immunotherapy considered. Even though this is a rare disease, questions relating to sequencing of therapy, including surgery and immunotherapy, should be evaluated by clinical trials.

## Data Availability

Not applicable.

## Abbreviations

CT: Computed tomography
FDG: Fluorodeoxyglucose
IHC: Immunohistochemistry
MiTF: Microphthalmia-associated transcription factor
OR: Overall response
PD1: Programmed programmed cell death protein 1
PD-L1: Programmed cell death ligand 1
PEComa: Perivascular epithelioid cell tumor
PET: Positron emission tomography
SUV: Standardized uptake value
XBRT: External beam radiation therapy
